# Deep-Sourced Fluids From a Convergent Margin Host Distinct Subseafloor Microbial Communities That Change Upon Mud Flow Expulsion

**DOI:** 10.3389/fmicb.2019.01436

**Published:** 2019-06-20

**Authors:** Scott A. Klasek, Marta E. Torres, Markus Loher, Gerhard Bohrmann, Thomas Pape, Frederick S. Colwell

**Affiliations:** ^1^Department of Microbiology, College of Science, Oregon State University, Corvallis, OR, United States; ^2^College of Earth, Ocean, and Atmospheric Sciences, Oregon State University, Corvallis, OR, United States; ^3^MARUM – Center for Marine Environmental Sciences and Department of Geosciences, University of Bremen, Bremen, Germany

**Keywords:** mud volcano, deep subsurface biosphere, microbial communities, methane oxidation, environmental gradient

## Abstract

Submarine mud volcanoes (MVs) along continental margins emit mud breccia and globally significant amounts of hydrocarbon-rich fluids from the subsurface, and host distinct chemosynthetic communities of microbes and macrofauna. Venere MV lies at 1,600 m water depth in the Ionian Sea offshore Italy and is located in a forearc basin of the Calabrian accretionary prism. Porewaters of recently extruded mud breccia flowing from its west summit are considerably fresher than seawater (10 PSU), high in Li^+^ and B (up to 300 and 8,000 μM, respectively), and strongly depleted in K^+^ (<1 mM) at depths as shallow as 20 cm below seafloor. These properties document upward transport of fluids sourced from >3 km below seafloor. 16S rRNA gene and metagenomic sequencing were used to characterize microbial community composition and gene content within deep-sourced mud breccia flow deposits as they become exposed to seawater along a downslope transect of Venere MV. Summit samples showed consistency in microbial community composition. However, beta-diversity increased markedly in communities from downslope cores, which were dominated by methyl- and methanotrophic genera of *Gammaproteobacteria*. Methane, sulfate, and chloride concentrations were minor but significant contributors to variation in community composition. Metagenomic analyses revealed differences in relative abundances of predicted protein categories between Venere MV and other subsurface microbial communities, characterizing MVs as windows into distinct deep biosphere habitats.

## Introduction

Seafloor mud volcanoes (MVs) release tens of teragrams of methane annually into the overlying water column (Kopf, 2003; Milkov et al., 2004; Sauter et al., 2006; Sahling et al., 2009; Pape et al., 2014; Mazzini and Etiope, 2017). Methane forms the basis of seafloor chemosynthetic ecosystems and is oxidized aerobically or anaerobically through microbial metabolism (Hanson and Hanson, 1996; Van Dover et al., 2003; Knittel and Boetius, 2009). MVs also expel fluids and mud breccia composed of clay-rich sediments with intermixed rock fragments, which can be sourced from several kilometers below the seafloor (Kopf, 2002; Mazzini and Etiope, 2017). High fluxes of methane and upward-directed fluid flow at active seafloor MVs can restrict the delivery of relevant electron acceptors, such as oxygen or sulfate, into the seafloor. This limits microbial capacity for methane oxidation (Niemann et al., 2006; Boetius and Wenzhöfer, 2013). Furthermore, anaerobic methanotrophs (ANMEs), the dominant consumers of methane in sediments (Reeburgh, 2007), have doubling times of up to 7 months *in vitro* (Nauhaus et al., 2007) and take years to dominate microbial communities *in situ* (Ruff et al., 2018). These limitations on biological methane removal support the argument that MVs represent potent sources of methane emission to the hydrosphere (Etiope and Milkov, 2004).

Microbial community changes in recently erupted muds of MVs are understood to occur on timescales of several years. In flows aged 1–2 years from the Håkon Mosby MV (Barents Sea), communities dominated by aerobic methanotrophic bacteria were identified as an initial successional stage (Ruff et al., 2018). Diffusion of electron acceptors, such as oxygen and sulfate, into fresh flows can be inhibited by upward transport of MV-derived mud breccia and reduced fluids (Niemann et al., 2006), but nevertheless is understood to drive a suite of redox-stratified microbial metabolisms that occur with increasing depth: aerobic oxidation of sulfide, sulfate reduction, anaerobic methane oxidation (AOM), and methanogenesis (Lin et al., 2018). Whether microbial communities across active MVs respond similarly to fluid expulsion is not known, given the considerable heterogeneity of fluid sources and compositions (Mazzini and Etiope, 2017). Additionally, how tectonically derived, deep-sourced MV fluids impact microbial community structure and potential activity remains poorly understood. Recently, the South Chamorro Seamount, a MV influenced by serpentinizing fluids derived from subduction at the Mariana convergent plate margin, has been described as an energetically favorable regime for microbial habitation: methanogens were recovered and identified, but methane from the fluids likely formed abiotically (Kawagucci et al., 2018). Considering the numerous challenges associated with drilling-based studies, microbial characterizations of deep subsurface systems are often limited to sites of fluid discharge into shallower horizons, often referred to as “windows to the deep biosphere” (Deming and Baross, 1993; Lee et al., 2015; Woycheese et al., 2015; Hoshino et al., 2017; Krauze et al., 2017).

Venere MV, in the central Mediterranean, is located within the Calabrian Accretionary Prism (CAP) (Panieri et al., 2013; Ceramicola et al., 2014), ∼50 km offshore Calabria at 1,600 m below seafloor (mbsf). Its active status is apparent through methane seepage along its caldera rim, the presence of visually distinguishable mud breccia deposits recently extruded from the western summit, and meter-scale changes in seafloor morphology documented by repeated bathymetric surveys between 2014 and 2016 (Loher et al., 2018a, 2018c). In mud breccia from the western summit, porewater chloride depletions to 20% of seawater values are consistent with smectite-illite clay dehydration reactions, and hydrocarbons show a thermogenic signature. These support fluid origin temperatures of 60–150°C that correspond to a depth between 3.5 and 7.5 km below the seafloor (Loher et al., 2018c), which may be within the 5–7 km sediment column overlying the basement of the CAP (Ferrucci et al., 1991; Voogd et al., 1992). Given that sedimentation rates at Venere MV and the nearby Crotone-Spartivento forearc basins range from 0.17 to 0.26 mm/year (Ceramicola et al., 2014; Loher et al., 2018a), the maximum ages of these recently extruded deposits are likely only a few years.

Using a suite of geochemical, 16S amplicon, and metagenomic sequence data, we sought to characterize microbial communities inhabiting deep-sourced mud breccia at Venere MV and to document changes in community composition associated with extrusion of flow deposits from the summit as they began to entrain seawater. We hypothesized that (1) both deep-sourced fluids and seawater-derived electron acceptors such as sulfate would exert a notable influence on microbial community structure; (2) methanotrophic taxa would be found in methane-rich mud breccia with increasing distance downslope of the Venere MV summit; and (3) gene content from MVs would be distinct from that of microbial communities inhabiting other subsurface environments.

## Materials and Methods

### Site and Core Descriptions

**FIGURE 1:**
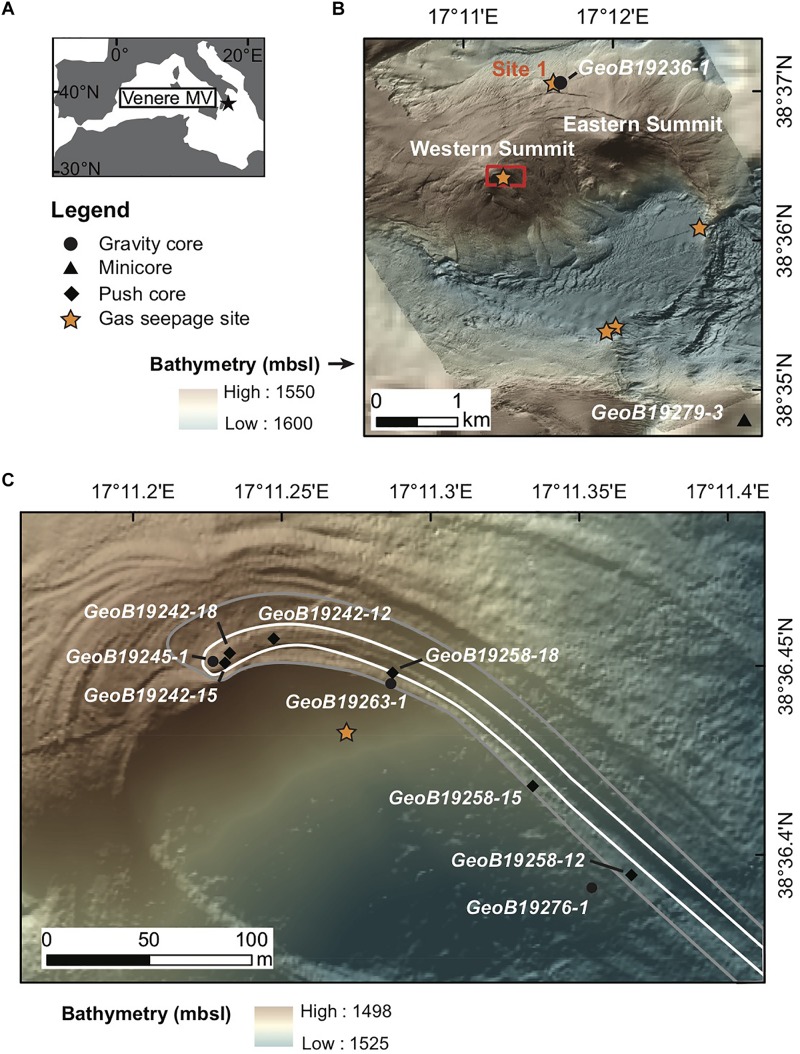
Venere mud volcano (MV) location, bathymetry, and sampling areas. **(A)** Venere MV lies offshore Calabria, Italy in the Mediterranean Sea. **(B)** AUV-derived bathymetry of Venere MV (1.6 m grid) showing Eastern and Western summits, ring faults along the caldera rim, five sites of observed gas seepage (orange stars), and locations of cores at seep site 1 and at a non-seep reference site to the southeast. Data from Loher et al. (2018a). **(C)** Zoomed-in view of the Venere MV western summit showing locations of gravity and push cores (circles and diamonds, respectively) collected for microbiological sampling. The extent of two recently extruded mud breccia flow deposits are shown with older deposits outlined in gray, underlying younger deposits delineated in white. Maps are modified with permission from Loher et al. (2018a).

Exposed mud breccia deposits at the western summit of the cone-shaped Venere MV (Figure 1) were visually investigated during multiple remotely operated vehicle (ROV) dives on MARUM cruise M112 aboard the R/V METEOR from November 29 to December 10, 2014. The most recently extruded deposits at the summit were gray in color and showed a rough surface texture, in contrast to deposits from previous mud breccia outflows, which were yellowish and characterized by a smooth texture. Sedimentological interpretation of cores from both flow deposits identified the material as mud breccia without any noticeable hemipelagic cover, and coring confirmed that the spatial extent of the flows extended up to 1.6 km downslope (Loher et al., 2018a, 2018c). Areas of methane seepage were hydroacoustically detected in the water column just below the western summit and at several locations around the caldera rim (Figure 1B) where the release of gas bubbles from the seafloor was visually confirmed (see initial descriptions by Loher et al., 2018b, 2018c).

### Sample Collection

For this study we targeted sites located at the summit, where freshened mud breccia had most recently been extruded. Gravity cores and a series of ROV-guided push cores obtained along a downslope transect of the mud flow deposits were collected and compared with a gravity core (GeoB19236-1) from gas seep site 1 (Figure 1B). Seep site 1 is located in the MV periphery on the rim of the caldera, where methane discharge is evident from hydroacoustic data and chemosynthetic communities typical of cold seep sites have been described (Loher et al., 2018b). This gravity core consists of mud breccia overlain by 2 cm of fine sandy sediment. In addition, a reference minicore (GeoB19279-3) retrieved southeast of the MV area (Figure 1B) was used as a background control. This core consisted of fine sand and hemipelagic sediment, lacking mud breccia or sediments affected by fluid seepage.

During this expedition, gravity core sediments were collected using a strategy developed to minimize the time between recovery and sampling of gas hydrate-rich sediments (Pape et al., 2010). Instead of using the common PVC liners, the core barrel was lined with a flexible polyethylene bag (film thickness 0.2 mm, R. Losch, Germany). Upon core recovery, the bag containing the sediment was removed from the barrel. The clear plastic allows for rapid inspection of the material. In contrast to conventional thick-walled PVC liners, the sediment can be sampled immediately by cutting through the bag with a sterile knife. Porewater, gas, and microbiology samples were collected less than 20 min after cores arrived on deck. Minicores and push cores were transferred to the ship’s laboratory and sampled in intervals of several centimeters by extruding the sediments from the plastic tube with a piston. Samples for DNA extractions and methane concentrations were collected from the same minicores and push cores, while parallel cores were sampled for porewater. Whole round core samples for DNA extraction were sliced using an ethanol-sanitized spatula and transferred to Whirl-Pak bags (VWR, Radnor, PA, United States) before being flash frozen in liquid nitrogen and stored at -80°C. To determine porewater methane concentrations, 3 ml of bulk sediment was transferred to a 20 ml glass vial filled with 5 ml NaOH, leaving 12 ml headspace. Rhizons (Seeberg-Elverfeldt et al., 2005) were used to draw porewater samples into acid-washed syringes over a period of 10 h. Porewaters were collected at room temperature, because bottom waters measured 18°C. Porewater samples for cation measurements were transferred to 1.5 ml acid-washed tubes and acidified with 20 μl ultrapure concentrated (65%) HNO_3_.

### Geochemistry

Salinity was measured onboard with a salinometer (Krüss Optronic, Hamburg, Germany) with a ±0.1 PSU precision, and calibrated daily against dilution of IAPSO standards with salinities of 9.989, 29.968, 34.993, and 38.022 PSU. Alkalinity was determined onboard by diluting a 0.5 ml porewater sample with 9.5 ml distilled water and stirring constantly in an open beaker while titrating with aliquots of a 0.01 M HCl standard until pH decreased below 4, normally between 3.9 and 3.5. The pH electrode (Hanna Instruments, Woonsocket, RI, United States) was calibrated with buffers of pH 4, 7, and 10. Alkalinity was calculated using the equation:

$$\text{Alkalinity}\left(\frac{\text{mol}}{\text{L}}\right)=\left[\left(v_{\text{HCl}}{}^{\text{*}}c_{\text{HCl}}\right)\text{​}-\text{​}10^{-\text{pH final}}{}^{*}\frac{v_{0}+v_{\text{HCl}}}{f_{{}^{\text{H+}}}}\text{​}+\text{​}10^{-\text{pH start}}{}^{*}v_{0}/f_{{}^{\text{H+}}}\right]/v_{0},(1)$$

where *v*_HCl_ is the volume (in ml) of HCl added to final pH, *c*_HCl_ is the molar concentration of HCl, pH_start_ and pH_final_ are the initial and final pH readings, *v*_0_ is the initial sample volume in ml, and *f*_H_ is the activity coefficient of H^+^ in seawater, taken as 0.79 (Culberson and Pytkowicz, 1973).

Dissolved methane in bulk sediment was analyzed onboard using an Agilent 6890N gas chromatograph (Agilent Technologies, Santa Clara, CA, United States) equipped with a capillary column connected with a flame ionization detector, following the same procedure as described in Pape et al. (2010). Reported concentrations of dissolved methane are *ex situ* concentrations, uncorrected for sediment temperature (Bunsen coefficient) and porosity. The high spatial heterogeneity of methane supply in the marine subsurface prevents an accurate determination of *in situ* porewater methane concentration, unless cores are analyzed at pressure (Milkov et al., 2003; Pape et al., 2011). Pressure cores from the Venere MV summit yielded average methane concentrations of 321 mmol/l, above the solubility of 120 mmol/l (Loher et al., 2018c), and two orders of magnitude higher than *ex situ* values reported here. This agrees with previous observations where ∼1% of methane by volume was recovered from gravity cores (Paull and Ussler, 2001). These loss estimates are corroborated by strong degassing and observed “moussy” textures of mud breccia recovered from gravity cores at the summit flow and at seep site 1 (Bohrmann et al., 2015). However, where our methane concentrations approach the solubility limit atmospheric pressure (∼1 mmol/l) we are not able to determine whether these measurements reflect *in situ* concentrations, or whether degassing occurred fully.

Sulfate and chloride concentrations were measured by ion chromatography using a Metrohm 882 Compact IC plus (Metrohm, Herisau, Switzerland) on a Metrosep A Supp 5 column. Analytical errors below 0.4% were reported for both ions. Linear interpolation was used to approximate chloride, sulfate, and alkalinity concentrations at depths where samples for microbiology were taken.

Lithium and boron samples were diluted 1:50, and potassium diluted 1:100, with 1% quartz-distilled nitric acid and run on a Leeman Labs Prodigy ICP-OES ion chromatograph using a radial viewing mode. IAPSO seawater and in-house solution standards were run every 11 samples and used to evaluate instrumental accuracy and precision. Detection limits were determined according to EPA method 200.7 (Environmental Monitoring Systems Laboratory, 1996). The limit of detection for potassium was 0.42 mM, while the limit of quantitation was 0.61 mM. High standards for lithium and boron were 10.2 and 327 μM, respectively.

### DNA Extraction for Amplicon Sequencing

DNA was extracted using a modified sodium dodecyl sulfate (SDS)-based DNA extraction protocol (Zhou et al., 1996). Other DNA extraction methods, such as Mobio PowerSoil kits (Qiagen, Hilden, Germany), or those detailed in Lever et al. (2015) produced much lower yields of genomic DNA that amplified less consistently. DNA extraction buffer was prepared as described in Zhou et al. (1996) and filtered through a 0.2-μm syringe filter. In a clean laminar flow hood, 0.3 g sediment, 550 μl extraction buffer, and 50 μl 20% 0.2 μl filtered-SDS were placed into autoclaved 1.5 ml internally threaded cryogenic vials (VWR, Radnor, PA, United States). Upon thawing, vials were vortexed at high speed for 5 min using a vortex affixed with a bead-beading adaptor (Qiagen, Hilden, Germany). Vials were sonicated in a room-temperature water bath sonicator at 40 kHz (Thermo Fisher Scientific, Waltham, MA, United States) in two pulses of 30 s each, separated by 90 s, and then incubated in a 65°C water bath for 1 h, inverting to resuspend flocculated sediment every 15–20 min. Vials were then centrifuged at 10,000 × *g* for 5 min to pellet sediment. Supernatants were combined with equal volumes of phenol–chloroform–isoamyl alcohol (25:24:1) in 2 ml heavy phase lock gel tubes (Quantabio, Beverly, MA, United States) and continuously inverted for 4 min. Tubes were spun down at 14,000 × *g* for 5 min, and aqueous layers carefully transferred to clean tubes with 2.5× the aqueous volume of ethanol and 1 μl glycoblue coprecipitant (Thermo Fisher Scientific, Waltham, MA, United States). DNA was precipitated at -20°C for 2 h, and then pelleted by centrifuging at 14,000 × *g* for 30 min at 4°C. Supernatant was carefully removed by pipetting and residual liquid was left to evaporate for 10–15 min. A subsequent salt wash was performed by adding 500 μl cold 70% ethanol and then centrifuging and removing the supernatant as before. Tubes were then placed in a Savant vacuum concentrator (Thermo Fisher Scientific, Waltham, MA, United States) at 55°C for up to 10 min to evaporate any remaining liquid. DNA pellets were resuspended in 50 μl nuclease-free water and stored at -20°C. Two DNA extraction blanks were conducted: one without any added sediment, and another with sediment that had been baked in an oven at 175°C for 2 h. DNA was measured with a Qubit Fluorometer and High Sensitivity Assay Kit (Thermo Fisher Scientific, Waltham, MA, United States) after extraction.

### 16S Library Amplification and Sequencing

16S amplicons were prepared following the Earth Microbiome Project Illumina protocol. V4 regions of bacterial and archaeal 16S rRNA genes were amplified in triplicate 25 μl reactions using universal 515-forward and 806-reverse primers (Caporaso et al., 2011) modified with dual-indexed Illumina sequencing adapters (Kozich et al., 2013). The thermal cycling protocol of Caporaso et al. (2011) was followed without modifications. After confirming amplification with agarose gel electrophoresis, triplicate PCR products were pooled and purified with a QIAquick PCR purification kit (Qiagen, Hilden, Germany). In addition to the DNA extraction blanks, a sample without any added template DNA was amplified and purified as a PCR negative control. Amplicon concentrations were quantified with a Qubit fluorometer (Thermo Fisher Scientific, Waltham, MA, United States) using the Qubit dsDNA high sensitivity assay kit and pooled in equimolar amounts. Illumina MiSeq V2 paired-end 250 bp sequencing was performed at the Oregon State University Center for Genome Research and Biocomputing (CGRB).

### 16S Amplicon Sequence Analysis

16S amplicon sequences were processed with version 1.39.3 of mothur (Schloss et al., 2009) following an established pipeline (Kozich et al., 2013). Reads were clustered into operational taxonomic units (OTUs) at a 97% similarity level and taxonomically classified using version 128 of the SILVA database (Quast et al., 2013). Singleton OTUs and contaminant genera were removed (for details on potential contaminants, see the section “Results”). Communities were rarefied to 356 reads, and relative abundances and metrics of alpha diversity (richness and Chao1, Shannon, and Simpson indices) were then calculated.

To compare beta diversity, a tree file containing the most abundant sequence from each of the 11,572 OTUs was constructed with Clearcut (Evans et al., 2006). A dissimilarity matrix was then calculated using weighted Unifrac distances (Lozupone et al., 2007). Principal coordinates (PCO) analysis was conducted in PRIMER7 (Clarke and Warwick, 2001) from the community dissimilarity matrix and a Euclidean-normalized environmental data matrix containing porewater chloride, methane, and sulfate concentrations corresponding to all samples. Distance-based linear modeling was used to evaluate the influence of these variables on community structure. Differences in community structure among sites and cores were evaluated using analysis of similarity (ANOSIM) (Clarke, 1993). Metastats (White et al., 2009) was used to determine whether individual OTUs showed patterns of differential abundance between communities based on chloride, methane, or sulfate concentrations and summit mud flow ages.

### DNA Extraction for Metagenome Sequencing

Metagenomic DNA from samples was extracted using the procedure described above, with modifications to account for the increased sediment volumes. 8.4 g from sample GeoB19242-15 at 2–3 cm, 4.8 g from sample GeoB19263-1 at 48–52 cm, and 31.2 g from sample GeoB19263-1 at 267–270 cm were used. Volumes of extraction reagents were scaled up according to sediment masses. Samples were placed in 15 or 50 ml conical tubes, and centrifuging steps were carried out at 4,500 × *g* for 10 min when pelleting sediment and 1,500 × *g* for 5 min when extracting with phenol–chloroform–isoamyl alcohol. Samples were precipitated at 4°C for 12 h with 0.6 volume isopropanol and 0.1 volume sodium acetate in 2 ml tubes. Pellets were pooled, desalted with ethanol as described previously, resuspended in nuclease-free water, and DNA was then purified with a Norgen CleanAll DNA/RNA clean-up and concentration kit (Norgen Biotek, Thorold, Ontario, Canada) according to manufacturer’s instructions for genomic DNA (>10,000 bp).

### Metagenomic Library Preparation, Sequencing, and Analysis

Metagenomic library construction and sequencing was conducted at the Josephine Bay Paul Center Marine Biological Laboratory (MBL), Woods Hole, MA, United States, using an established protocol available online^1^. To add to the three samples described here, fastq files from seven additional metagenomes derived from recent mud flows of the Håkon Mosby MV (Ruff et al., 2018) were downloaded from NCBI and analyzed in parallel. The ends of low-quality fastq sequences were removed using version 1.0-r72-dirty of the seqtk trimfq tool^2^. Paired reads were assembled with Megahit v1.1.1-2-g02102e1 (Li et al., 2015) and annotated in ShotMAP v1.1 (Nayfach et al., 2015) against the clusters of orthologous group (COG) protein family database (Galperin et al., 2015) using a classification threshold score of 42.8 and a coverage-based abundance calculation strategy. This classification method was more accurate than other pre-2018 pipelines in quantifying gene family abundances using the COG database (Franzosa et al., 2018). Alpha diversity statistics were obtained across all three Venere and seven Håkon Mosby MV metagenomes using the ShotMAP script compare_shotmap_samples.pl. Percent abundances of reads annotated to each COG functional category were summed across samples. Reads annotated to multiple COG categories were omitted and percent abundances re-normalized to allow for comparison against other publicly available metagenomes from subsurface ecosystems annotated using the COG database. This information was obtained from the MG-RAST and IMG/M metagenome databases (Markowitz et al., 2014; Keegan et al., 2016).

## Results

To investigate microbial community changes during the extrusion and downslope transport of recently exposed, chloride-depleted mud breccia originating from the marine subsurface and the influence of seawater entrainment in such sediments, we collected samples for geochemical and microbial analyses from Venere MV offshore Calabria, Italy (Figure 1A). In the following, we describe results of DNA extractions/quality controls, geochemical trends that characterize Venere MV summit flow deposits, and then integrated geochemical and microbial community observations. We finally compare gene content from Venere MV metagenomes with that of another marine MV and additional marine subsurface environments. Table 1 includes relevant information from all cores organized by their sampling locations.

**TABLE 1:** Sampling sites and additional information for all cores in this study.

**Sampling site**	**Core (GeoB)**	Core type^*^	**Flow age**	**Specific location**	**Metagenomes**	**Figure**
Venere MV summit flow deposits	19245-1	GC	Younger	Summit	NA	4
	19263-1	GC	Older	80 m downslope	48 and 267 cmbsf	4
	19276-1	GC	Just outside older	150 m downslope	NA	4
	19242-15	PC	Younger	5 m downslope	2 cmbsf	5
	19242-18	PC	Younger	10 m downslope	NA	5
	19242-12	PC	Younger	30 m downslope	NA	5
	19258-18	PC	Older	80 m downslope	NA	5
	19258-15	PC	Older	110 m downslope	NA	5
	19258-12	PC	Older	150 m downslope	NA	5
Site 1 seep	19236-1	GC	NA	NA	NA	4
Reference	19279-3	MC	NA	NA	NA	5

GeoB numbers, coring equipment, mud flow ages, spatial relation to the Venere MV summit, cores/depths of metagenomes from Venere MV, and figures showing geochemistry and microbial community data. ^*^GC, gravity core; PC, push core; MC, minicore.

### Yield and Controls for DNA and 16S Amplicon Sequence Analysis

DNA extraction yields from summit mud breccia sediments ranged across several orders of magnitude, from <0.12 to 145 ng per g bulk sediment. Yields were lowest in samples taken from depths below 40 cm in the summit gravity core, and higher in surface samples <10 cm below seafloor (cmbsf) and throughout gravity core GeoB19263 taken near seep site 3 just below the western summit (Figure 1C). Genomic DNA yields from push cores of the newer summit flow deposit (GeoB19242) were mostly below 2 ng per g, and push cores from the older flow further downslope showed increasing yields with distance from the summit (Supplementary Figure S1). Mud breccia samples with added *Escherichia coli* genomic DNA recovered only 8.3% of the DNA compared to extractions with gDNA and no mud breccia. Therefore, in addition to the evident low biomass within these samples, sorption of DNA to clayey materials also likely reduced recovery. Faint amplification was observed in one DNA extraction blank, but not in another with baked sediment added to it, which suggests the mud breccia may contain PCR inhibitors. In addition, several samples intended for 16S amplicon sequencing failed to amplify.

The low amounts of extracted DNA recovered from Venere MV samples necessitated extra scrutiny in identifying and removing potential contaminant sequences. Nineteen bacterial genera present at higher abundances in the three blanks than in 68 samples (α = 0.05) were removed, as were four genera previously identified as common laboratory or reagent contaminants in low-biomass samples (Salter et al., 2014). The removed genera (listed in Supplementary Table S1) comprised six bacterial phyla and 81% of the sequences identified in blanks, but only 2.7% of Venere MV communities overall. Bacterial and Archaeal genera that were plotted in figures include the 13 genera that comprised >1% of all sequences in the dataset, and an additional six genera that were each over 0.3% of all sequences and were over 10% of any single community. Altogether, these 19 genera made up 72.5% of all sequences in the dataset.

### Geochemical Signature of Venere MV Summit Flow Deposits

**FIGURE 2:**
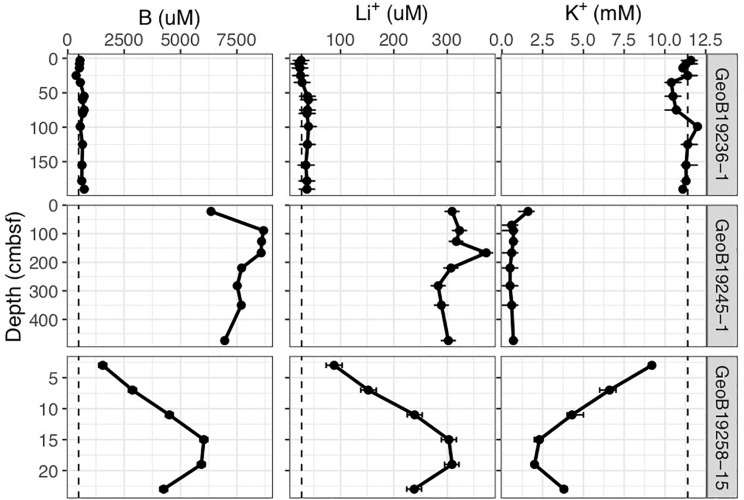
Lithium, boron, and potassium concentrations in porewater of freshly extruded Venere MV mud breccia flows. Cores include a gravity core from Seep Site 1 away from the fresh mud breccia deposits (GeoB19236-1), a gravity core from the mud breccia extrusion site at the summit (GeoB19245-1), and a push core 110 m downslope (GeoB19258-15). Dashed lines indicate seawater values, and confidence intervals represent standard deviations.

**FIGURE 3:**
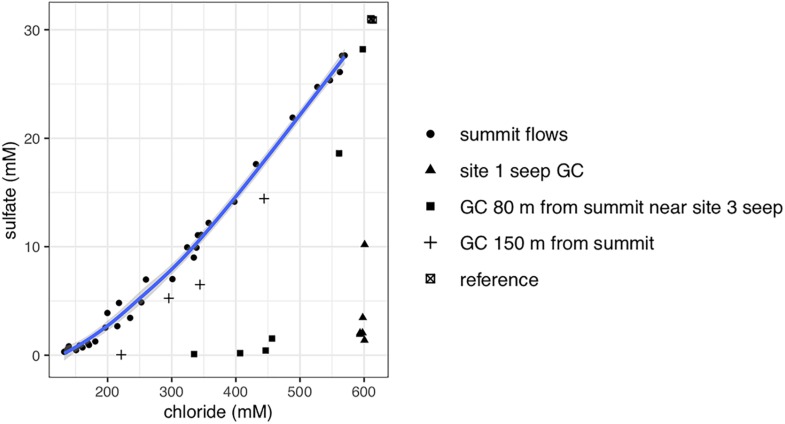
Biplot of porewater chloride and sulfate concentrations from Venere MV depths sampled for microbial communities. Summit flow deposits representing mixed fluids are depicted as filled circles, while other symbols represent individual cores. Downslope gravity cores (GCs) are shown as plus symbols or filled squares. Cores located away from the summit flow deposits are shown as filled triangles or crossed squares. The shaded line around summit samples is a 95% confidence interval calculated by locally estimated scatterplot smoothing.

**FIGURE 4:**
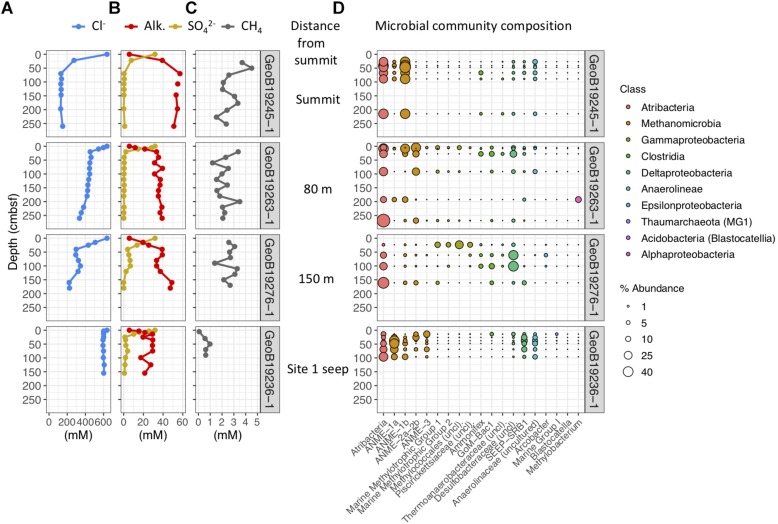
Geochemical and microbial community data from mud breccia gravity cores at the summit, 80 m downslope, 150 m downslope, and the site 1 seep north of the summit flows, ordered from top to bottom. **(A)** Porewater chloride, **(B)** alkalinity and sulfate, and **(C)** methane concentrations. **(D)** Percent abundances of dominant bacterial and archaeal genera within microbial communities, colored by taxonomic classes to which they belong.

Gravity and push cores (GeoB19245-1 and 19258-15, respectively) collected at the Venere MV summit and downslope flow deposits show elevated concentrations of lithium and boron, and a depletion in potassium at depths just centimeters below seafloor (Figure 2). These contrast markedly with trends seen in the gravity core from seep site 1 (GeoB19236-1), where these constituents reflect concentrations found in bottom waters. In samples from Venere MV summit flow deposits where microbial community samples were collected, porewater chloride and sulfate concentrations show a positive linear relationship (Figure 3). Chloride decreases with depth at the summit (Figure 4A). Because chloride changes occur at depths where lithium, boron, and potassium also deviate from seawater values, we interpret the coinciding decreases in chloride as a distinct geochemical transition between deep-sourced and seawater-influenced end-members.

### Fluid Geochemistry and Microbial Community Diversity

Results are organized according to sampling areas or depths. First we show geochemical and microbial community data from gravity cores of Venere MV summit mud flow deposits and methane seep site 1, an area uninfluenced by recent mud breccia extrusion. These cores reach over 1 mbsf. We then present comparable data from push cores from the summit flow deposits and a reference site (without evidence of mud breccia extrusion or fluid seepage) that detail depths up to 20 cmbsf.

#### Gravity Core Geochemistry and Microbial Communities

Porewaters freshen markedly in gravity cores from the Venere MV summit and in cores GeoB19263-1 and GeoB19276-1, collected 80 and 150 m downslope along the mud flow (Figure 4A). In contrast, the site 1 seep core (GeoB19236-1) shows no evidence of porewater freshening even though sediments are mainly comprised of mud breccia. The deep-sourced fluids in summit gravity cores are highly alkaline (40–60 mM) and depleted in sulfate, as are fluids from the site 1 seep (Figure 4B). Low sulfate/chloride ratios in gravity core samples from mud flow deposits downslope of the summit suggest the occurrence of microbial sulfate reduction (Figure 3). The accumulation of methane below the sulfate reduction zone at seep site 1 (Figure 4C) shows a sulfate–methane transition zone that suggests the presence of AOM. Methane concentrations in summit gravity cores range from 2 to 4 mM, but do not decrease with depth as conspicuously. Microbial communities at all depths of the summit gravity core show consistent relative abundances of Atribacteria, ANME-1b, and Anaerolineaceae (Figure 4D). Other methanotrophs are present in two cores 80 and 150 m downslope of the summit: ANME-2a-2b in GeoB19263-1, and several genera of aerobic methanotrophic Gammaproteobacteria at 24 cmbsf in GeoB19276-1. Clostridia and Desulfobacteraceae, potential sulfur cyclers, are also more abundant in communities from downslope gravity cores. In contrast, the seep site 1 microbial community shows high abundances of several ANME clades throughout the core. Sulfate-reducing bacteria (SRB) SEEP-SRB1, which often associate with ANME to mediate AOM (Knittel et al., 2003), are also present in high abundances.

#### Push Core and Minicore Geochemistry and Microbial Communities

**FIGURE 5:**
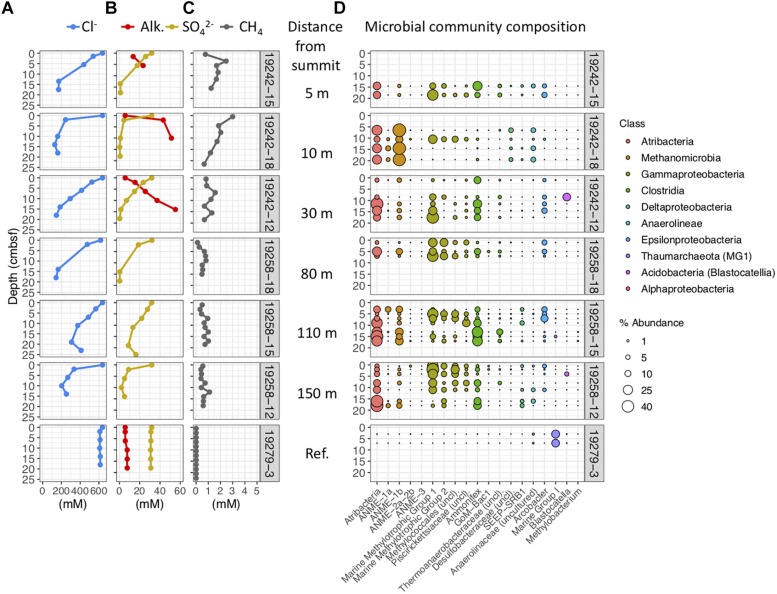
Geochemical and microbial community data from mud breccia push cores collected near the summit, ordered by increasing distance downslope from top to bottom, and a reference minicore southwest of the flow deposits. The first three cores are collected from within the young flow at the summit and 5 and 10 m downslope, followed by three from 80, 110, and 150 m downslope within the older flow. **(A)** Porewater chloride, **(B)** alkalinity and sulfate, and **(C)** methane concentrations. **(D)** Percent abundances of dominant bacterial and archaeal genera within microbial communities, colored by taxonomic classes to which they belong.

ROV-guided push cores taken from Venere MV summit and downslope from the mud breccia extrusion site also show significant freshening in porewater with chloride values of 200 mM measured only a few centimeters below seafloor, whereas the reference minicore GeoB19279-3 does not (Figure 5A). All cores, with the exception of the reference, show decreasing sulfate and increasing alkalinity with depth (Figure 5B). Methane concentrations in mud flow deposit cores were higher closer to the summit, but still exceeded 1 mM at 150 m downslope and generally did not drop off with depth (Figure 5C). Methane in the reference core porewater was detected in nanomolar amounts. As in the gravity cores, push core communities contained high abundances of Atribacteria and ANME-1b, particularly in core GeoB19242-12 near the summit (Figure 5D). However, methylotrophic Gammaproteobacteria were the most dominant community members in cores from the older mudflow sequence downslope of the summit. The top four OTUs from this group matched most closely to the genus *Methylomonas*, which are obligately aerobic type 1 methanotrophs capable of fixing nitrogen (Lidstrom, 2006). High abundances of an OTU belonging to *Ammonifex* and most similar to *Thermodesulfitimonas autotrophica*, an anaerobic chemolithoautotroph (Slobodkina et al., 2017), were present at similar or slightly deeper depths. The most abundant OTU, a marine group 1 methanotroph, is most closely related to *Methylomonas methanica* (Boden et al., 2011). Bacterial and archaeal genera present in high abundance in summit flow communities were not present in shallow sediments of the reference site, which instead contained high abundances of Marine Group I Thaumarchaeota (Figure 5D). However, these communities were characterized by much higher alpha diversity than those from all other cores, with Chao1 indices of 606–782 compared to 153 or lower in mud breccia communities.

### Community Diversity and Variation With Porewater Geochemistry

**FIGURE 6:**
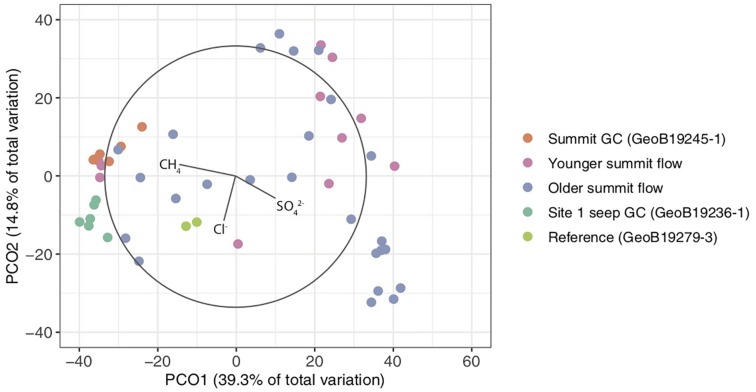
Principal coordinates (PCO) analysis plot of microbial communities from Venere MV summit deposits, seep site 1, and a non-seep reference. Vectors represent porewater geochemical concentrations that correlate with differences among microbial communities.

A PCO ordination analysis was used to examine patterns of beta diversity among Venere MV microbial communities and their relation to porewater geochemical parameters (Figure 6). Two PCOs captured 54.1% of the community variance. Communities from Venere MV summit deposits showed the highest beta-diversity, though a high degree of similarity was observed in communities from the summit gravity core. ANOSIM tests indicated differences in community structure between samples from older and younger summit mud breccia deposits, and between most cores (34 of 55 pairwise comparisons). However, when summit gravity core communities were reclassified as a distinct group instead of as younger mud flows, this difference disappeared (Supplementary Table S2). For this reason, and because they showed high similarity, summit gravity core communities were colored separately on the ordination (Figure 6). It is noteworthy that communities from seep site 1 and from a reference core were distinct from those inhabiting mud breccia flow deposits at or near the summit (Supplementary Table S2). Communities from these deposits recently extruded from kilometer-scale depths may require time to adapt to physical and/or geochemical conditions of a shallower marine subsurface environment.

Porewater sulfate, methane, and chloride concentrations were all significant drivers of community structure (*p* ≤ 0.006), and, respectively, accounted for 8.6, 7.6, and 6.4% of the difference in community structure (22.6% of the total). Methane and sulfate vectors point in opposite directions on the PCO (Figure 6), reiterating contrasting observations of methane-rich, sulfate-depleted fluids in summit and seep site 1 gravity cores compared to surface mud breccia deposits which have begun to entrain sulfate-rich seawater (Figures 4, 5). On the other hand, direct trends between porewater chloride and community composition are not conveyed in the ordination (Figure 6), which is likely obscured by the high beta-diversity among summit flow communities.

Several abundant OTUs in Venere MV summit flows are present in communities at different abundances based on mud flow age, and methane or chloride concentrations (Supplementary Figure S2). One aerobic methanotroph belonging to *Methyloccocales* was more dominant in older summit flow communities. Another, classified to Marine Methylotrophic Group 1, constituted nearly a quarter of the communities where methane was below 0.5 mM, suggesting it was active in removing methane from pore fluids. Atribacteria dominated methane-rich sediments, and two OTUs belonging to *Desulfobacteraceae* were more abundant in older deposits where methane concentrations still exceeded 2 mM. ANME-1b was the only OTU showing a preference for deeply sourced fluids with low chlorinity (Supplementary Figure S2C). This ANME was presumably dead, dormant, or outcompeted by other populations as mud breccia was extruded from the Venere MV summit and exposed to a more oxidizing environment.

### Metagenomes From Venere and Other MVs

**FIGURE 7:**
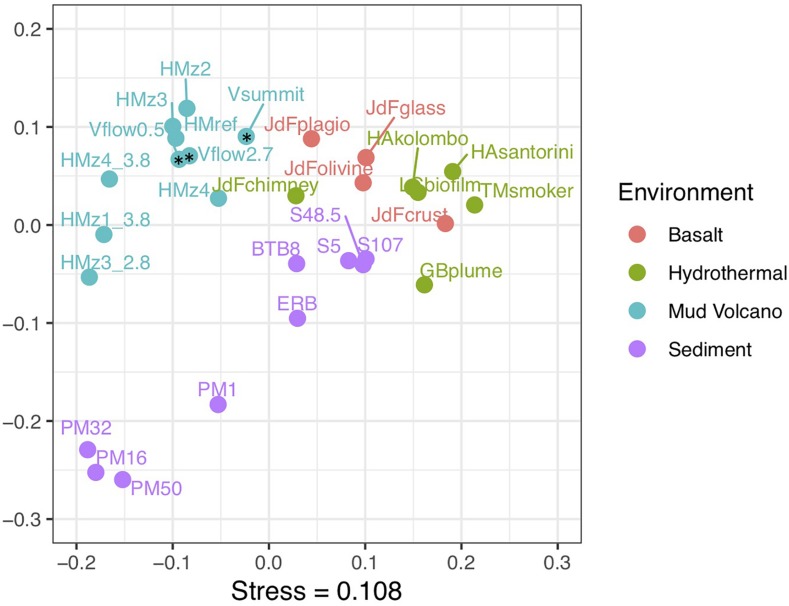
Non-metric multidimensional scaling (NMDS) ordination of COG functional category percent abundances among publicly available metagenomes from diverse marine subsurface environments. Venere MV samples are indicated by asterisks. V, Venere MV; HM, Håkon Mosby MV; JdF, Juan de Fuca; HA, Hellenic Arc; TM, Tahi Moana; GB, Guaymas Basin; PM, Peru Margin, ERB, Eel River Basin. Where indicated, numbers at ends of labels represent depths below seafloor in meters. Additional information is supplied in Supplementary Table S4.

Metagenomes were analyzed from three samples at Venere MV, a surface mud flow deposit near the Venere MV summit, and 50 and 267 cmbsf from gravity core GeoB 19263-1, 80 m downslope. COG category abundances from Venere MV metagenomes were similar to those of seven others from Håkon Mosby MV, off Norway in the Barents Sea (Ruff et al., 2018). The diversity of COGs among all MV metagenomes did not vary significantly. However, these two MVs together showed a distinctly different composition of COG categories than metagenomes belonging to hydrothermal vent, basaltic crust, or sediment ecosystems (Figure 7 and Supplementary Table S3). Supplementary Table S4 contains additional information for other metagenomes compared, which either displayed COG category information in-text or online in MG-RAST or IMG/M databases. In particular, MV metagenomes showed higher abundances of reads classified to defense mechanisms (to combat viral attack) than the other three environments (Supplementary Figure S3). In addition, higher content in the categories of cell wall/membrane/envelope biogenesis, translation, and posttranslational modification came at the expense of energy production/conversion and amino acid transport and metabolism. These broad-level differences may hint at specialized adaptations that allow life to persist in a distinct deep subsurface environment.

## Discussion

Mud volcanoes emit large quantities of methane into the oceans, contain fluids generated at depths from clay dewatering and convergent margin activity, and host methane-oxidizing chemosynthetic communities (Corselli and Basso, 1996; Fitts and Brown, 1999; Kopf, 2002, 2003; Niemann et al., 2006). In this study, we sought to investigate linkages between methane, deep-sourced fluids, and microbial community structure and gene content at a subseafloor MV to an unprecedented extent by characterizing microbial communities in freshly exposed mud breccia deposits at Venere MV within a geochemical context as they were extruded onto the seafloor. First we consider the geochemical nature of the deep fluids, then follow with a discussion of microbial gene content and metabolic potential, and finally place microbial community changes within the context of this transitional environment.

### Deep-Sourced Fluids

As interpreted previously by Loher et al. (2018c), the decrease in pore water chloride at Venere MV summit and mud flow deposits appears to be the result of the upward advection and transport of deep-sourced fluids, which appear to be generated not from gas hydrate decomposition but by smectite-illite clay mineral dehydration reactions (Loher et al., 2018c), which occur at temperatures between 60 and 150°C (Kastner et al., 2014). The estimated regional heat flow gradient of 0.020°C/m and the thermogenic hydrocarbon signature documented at the summit of Venere MV point to a fluid source at depths of 3–7.5 km below the seafloor (Loher et al., 2018c).

Boron enrichment in pore waters typically results from clay particle desorption during mineral dehydration reactions or leaching from basalts at high (>150°C) temperatures (Seyfried et al., 1984; Kopf and Deyhle, 2002), processes which have repeatedly been documented by fluid analyses along convergent margin subduction zones (You et al., 1993, 1995). Lithium may also be released from sediments and basalts at temperatures ranging from 51 to 350°C (You et al., 1995; James et al., 2003), which may point to fluid sources located deeper than smectite-illite transition depths, as in MVs from the Nankai accretionary wedge (Nishio et al., 2015). At the Venere MV summit, porewater boron concentrations of up to 13 mM rank among the highest ever documented at MVs, which typically range below 5 mM (Kopf and Deyhle, 2002). Porewater lithium values of several hundred micromolars at Venere MV are comparable in magnitude to the MVs of the Barbados convergent margin (Martin et al., 1996), and exceed those of the Kumano MVs of the Nankai wedge (Nishio et al., 2015). Boron and lithium enrichments have also been noted to infer fluid mobilization depths of 4–6 km below seafloor in the active Carmen MV in the western Mediterranean (López-Rodríguez et al., 2019). Potassium concentrations below 1 mM in Venere MV pore fluids are without precedent, though values as low as 2.4 mM in MVs of the Barbados margin have been interpreted as incorporation of K^+^ into the clay mineral illite during smectite diagenesis (Martin et al., 1996). This phenomenon has been noted at other sites including the Ulleung basin (Kim et al., 2016) and Canadian Shield sedimentary deposits (Bottomley and Clark, 2004). Together, the magnitudes of chloride and potassium depletions at Venere MV point toward considerable fluid generation from clay dewatering during smectite-illite clay mineral transformations at depth.

Below 1 m, porewater chloride (Figure 4A), potassium, boron, and lithium (Figure 2) in the Venere MV summit gravity core do not change appreciably. This suggests that below this horizon, there is no admixture with seawater but that active upward advection of deeply sourced (>3 km) fluids maintained their highly altered fluid composition to depths as shallow as 50 cmbsf. The nonlinear nature of these profiles (Figures 4A,B) evinces a system out of steady-state conditions, wherein seawater has only begun to diffuse downward into the mud breccia to depths of 50 cm or less.

### Distinct Microbial Gene Content at Venere MV Compared With Other MVs

To compare microbial communities with the potential nature of the fluid regime, we use low chlorinity (<200 mM) in Venere MV summit and mud flow deposits as a proxy for deep-sourced fluids. Despite the considerable geochemical transition from deep-sourced to seawater-influenced fluids in Venere MV flow deposits, chloride concentration only explained 6.4% of the difference among microbial communities. This would suggest that communities in recently extruded mud breccia are affected only minimally by these deep fluids, or have not yet had the time to shift as they begin to mix with seawater and electron acceptors therein. Only one OTU, an ANME-1b, changed noticeably with chloride concentration (Supplementary Figure S2C); it may thus be inactive or outcompeted by other taxa considering that doubling times for anaerobic methane oxidizers are over a month, and that years may be required before they become dominant within communities (Girguis et al., 2005; Ruff et al., 2018).

Despite the low influence of deep fluids on community structure, clear patterns are noted in mud flow deposits with increasing distance from the extrusion site. The increased percent abundances of methyl- and methanotrophic Gammaproteobacteria, Clostridia, *Ammonifex*, and unclassified *Desulfobacteraceae* (Figures 4, 5) likely reflect new growth instead of a decline in actual numbers of competing taxa. Higher abundances of these taxa in downslope cores may reflect proliferation in response to changes in physical conditions (such as changes in temperature and pressure), geochemical conditions (presence and nature of electron acceptors), and time after mud flow expulsion from the center conduit, which may allow methane to diffuse upward through flow deposits and possibly into the water column.

Microbial groups present in mud breccia extruded from the main conduit at the western cone of Venere MV presumably have adaptations to high temperature environments, or are able to persist in a dormant state under considerable energy limitation for extended timescales (Hoehler and Jørgensen, 2013). Cell generation times in sediments can increase at least two orders of magnitude below sulfate–methane transition zones (Starnawski et al., 2017). The high degree of similarity between Venere MV communities throughout summit gravity core samples across the transition from deep-sourced to seawater-influenced fluids (Figures 4, 6) suggests that community changes do not occur instantly, and that the few taxa therein represent an end-member deep-sourced microbial community signature from within the MV conduit. Thermophilic ANME-1 clades have been described at hydrothermal vents and sediments impacted by hydrothermal fluids (Teske et al., 2002; Brazelton et al., 2006), while Atribacteria are understood to disperse through mud volcanism (Hoshino et al., 2017). The functional and genomic potential within the class Anaerolineae is still poorly understood, but some members are considered to be anaerobic fermenters that show evidence of cellulolytic activity (Xia et al., 2016). Additional investigations are needed to determine whether community members present in deep-sourced mud breccia constitute live and potentially active populations.

Currently, only members of the bacterial phylum Firmicutes are understood to form endospores (Fritze, 2004), and Clostridia are the only class of these present in significant amounts at Venere MV communities. In particular, *Thermoanaerobacteraceae* and *Ammonifex* contain hyperthermophilic chemolithotrophic members isolated from hot springs that employ hydrogen or formate as electron donors and several sulfur compounds as acceptors (Miroshnichenko et al., 2008; Slobodkina et al., 2017). These groups are most prevalent in shallow push core sediments where sulfate is present (Figure 5), yet the sulfate in these deposits is consistent with a mixing ratio with deep-sourced fluids and seawater as end members, suggesting an absence of ongoing sulfate reduction (Figure 3).

Microbial communities at MVs display high variability, though several keystone OTUs involved in methane and sulfur metabolism occur in these systems, particularly members of ANME, Delta-, and Gammaproteobacteria (Pachiadaki and Kormas, 2013). Methanotrophic communities are restricted to surface sediments at the center of the active Håkon Mosby MV, which is characterized by a dynamic gas hydrate system (Pape et al., 2011). ANMEs at Håkon Mosby MV are active in peripheral zones, where lower rates of deeply sourced fluid flow allow sulfate to permeate deeper into the sediment column (Niemann et al., 2006). Studies of recent deposits at this location identified additional populations similar to those found at Venere MV, including Atribacteria and Chloroflexi associated with the subsurface conduit, and sulfate reducers and sulfur oxidizers in surface-exposed mud deposits (Ruff et al., 2018). In addition, high abundances of Methylococcales (up to 20%) have been found to characterize communities of active MVs in the Mediterranean compared to inactive ones nearby (Coelho et al., 2016). In contrast to active methanogens described at other MVs (Lazar et al., 2012) or deep biologically sourced methane described at other accretionary margins (Ijiri et al., 2018), the low abundance of sequences belonging to canonical methanogens (0.7%) and thermogenic isotopic signatures of hydrocarbons at the summit of Venere MV suggests that microbial methane production is negligible.

Based on preliminary comparisons with other marine subsurface ecosystems, the distinct gene content in MV metagenomes (Figure 7) likely reflects functional adaptations in community members that may not be endemic to MVs, but are nevertheless highly abundant therein. Elevated percentages of defense mechanism COGs in MV metagenomes are suggestive of diversified adaptations against viral attack (Makarova et al., 2013), though viral diversity may select against certain defense elements (such as CRISPR-Cas systems) from the genome (Weinberger et al., 2012). Viruses can outnumber bacterial and archaeal cells in subsurface environments by one to two orders of magnitude (Engelhardt et al., 2014; Nigro et al., 2017), and as such, their influence on microbial community dynamics and evolution within MVs should be topics of future investigation.

Compared to basalts and hydrothermal environments, the higher percentages of COGs related to translation, ribosomal structure, and biogenesis in MVs suggest smaller average genome sizes (Konstantinidis and Tiedje, 2004). The gain, loss, or modification of cell membrane genes in populations of *Sulfurovum* has been implicated to be adaptations to hydrothermal environments (Anderson et al., 2017); these forces could also explain high percent abundances of cell membrane COGs in MVs. With the exception of amino acids, the similar abundances of metabolic COG categories between MVs and other subsurface environments could imply that metabolic gene content across subsurface environments is somewhat homogenous at a broad level of classification. Further analyses of MV metagenomes should continue characterizing metabolisms involved in biogeochemical cycles, and pay particular attention to specific adaptations to temperature, pressure, or the unique geochemistry of deep-sourced fluids these microbial genomes could hold.

### Community Changes Upon Mud Breccia Expulsion on a Transitional Environment

Microbial community changes have been characterized across transitional subsurface environments in many settings. Increased diversity observed in aging basalt outcrops has been explained by geochemical changes, such as the accretion of organic matter and allochthonous minerals, that could support a wide variety of metabolisms (Lee et al., 2015). In contrast (Woycheese et al., 2015), observed a decrease in diversity in serpentinizing seep fluids after they were exposed to the atmosphere and a select few genera became dominant. It took around 2 years for alpha diversity to increase in freshly exposed muds at Håkon Mosby MV, despite available electron donors and acceptors (Ruff et al., 2018). Patterns of alpha diversity in Venere MV mud flow deposits are not apparent, and thus may reflect a similar time dependency.

The observation that Venere MV mud flow microbial community composition was influenced by methane and sulfate availability is suggestive of energy limitation, which has also been found to structure communities in deep sediments of the South China Sea (Graw et al., 2018). Preliminary IODP data from Expedition 370 found that subsurface temperatures above 65°C can drastically restrict cell numbers (Heuer et al., 2017). Incomplete heat flow data from shallow Venere MV deposits prevented us from determining the influence of temperature on community composition. Decreasing pressure as fluidized muds are transported upward may represent another physical factor that potentially influences microbial community composition (Bartlett et al., 2007; Myka et al., 2017). Differences observed between communities from the Venere MV summit gravity core and nearby downslope deposits may indicate additional geochemical or physical forces at play, though community assembly may be stochastic to some extent (Stegen et al., 2012).

Similar to microbial communities in extruded mud breccia flows of the Håkon Mosby MV (Ruff et al., 2018), we observed increases in SRB (*Desulfobacteraceae* and SEEP-SRB1) with time and distance from the MV summit, mirroring trends in genomic DNA concentrations (Supplementary Figure S1). These changes were particularly evident in cores with high porewater chloride but low sulfate, which points toward ongoing microbial sulfate reduction (Figure 3). Populations of ANME did not change or increase in abundance, though ANME-2a-2b were one notable exception, present in downslope gravity cores and also at the more mature seep site 1 (Figure 4D). We postulate that mud breccia deposits closest to the summit (<80 m) are too young for ANME populations to have developed. If community successional patterns align with those described by Ruff et al. (2018), these flows were likely extruded less than 2 years ago, and push core communities dominated by aerobic methanotrophs may be even less than a year old. This is in line with findings by Loher et al. (2018a) who documented morphological changes near the western summit of Venere MV between 2014 and 2016 that support its active status.

Nevertheless, the high abundances of aerobic methyl- and methanotrophic Gammaproteobacteria in recently exposed MV surface sediments are somewhat unexpected. These groups dominate downslope push cores that are lower in methane than others closer to the summit (Figure 5C), but they are highly abundant >20 cmbsf into the low-chloride and presumably oxygen-free mud breccia fluids, inhabiting the same depths as obligate anaerobic genera such as *Ammonifex*. Although Gammaproteobacteria have been shown to dominate bioturbated sediments (Chen et al., 2017) and maintain endosymbioses with Siboglinid polychaete worms (Thornhill et al., 2008), no macrofauna were found in Venere MV summit flow deposits. One possible mechanism of delivering oxygen from bottom water into the mud breccia would be via advection through the plated and fractured surface structure of the most recently extruded mud breccia. Testing this hypothesis would require oxygen measurements, which are presently not available. AOM by bacteria is not without precedent: *Methylomirabilis*, of the NC10 phylum, uses nitrite to generate oxygen and oxidize methane using a particulate methane monooxygenase homologous to those found in aerobic methanotrophic bacteria (Ettwig et al., 2010). Although the typically clay-rich matrix composition of mud breccia would limit diffusion rates, the low biomass in Venere MV summit mud flow deposits may not consume oxygen very quickly. Bottom seawater penetration into recently extruded mud breccia could also explain the delivery of methylotrophic Gammaproteobacteria into shallow mud breccia depths, as these groups normally persist in bottom waters (Tavormina et al., 2010) or at the sediment–water interface, and were not detected in deep sediments from the summit GC. Additional constraints on mud flow dynamics, pore fluid movement, and geochemical interfaces are needed to better understand how microbial communities respond to a highly dynamic environment.

## Conclusion

The active Venere MV, located on the CAP offshore Italy, emits fluids and mud breccia sourced from kilometer-scale depths within sediments that are depleted in sulfate and chloride with respect to seawater. Microbial communities inhabiting summit mud flow deposits are distinct from those at reference and peripheral methane seepage sites. Downslope mud breccia communities contain several clades of aerobic methyl- and methanotrophic Gammaproteobacteria, while ANMEs and SRB develop in older mud breccia deposits further from the summit. Spatiotemporal succession patterns of similar communities from another MV suggest that Venere MV flows may have been extruded less than 2 years ago. Microbial gene content from both of these MVs is distinct from that of other marine subsurface habitats, and likely reflects adaptations to a unique environment.

## Data Availability

Raw (fastq) sequence data corresponding to this study deposited under NCBI Short Read Archive BioProject PRJNA531342.

## Author Contributions

SK designed the study, conducted lab work, analyzed data, interpreted results, and wrote the manuscript. MT collected samples, provided geochemical data and analysis, and helped edit the manuscript. ML provided mapping data and geophysical and geochemical interpretations. GB served as a chief scientist on the research cruise to the field site and provided additional geochemistry data. TP contributed methane measurements and additional interpretations. FC helped with data interpretation and manuscript editing.

## Conflict of Interest Statement

The authors declare that the research was conducted in the absence of any commercial or financial relationships that could be construed as a potential conflict of interest.

## Footnotes

1 https://vamps.mbl.edu/resources/faq.php#methods

2 https://github.com/lh3/seqtk
