# Supplementary material for: Deep-Sourced Fluids From a Convergent Margin Host Distinct Subseafloor Microbial Communities That Change Upon Mud Flow Expulsion
Source: Front Microbiol. 2019 Jun 20;10:1436. doi: 10.3389/fmicb.2019.01436 (PMC6596357; doi:10.3389/fmicb.2019.01436)
Supplement: Supplementary file 1 [file Data_Sheet_1.docx]

**Figure S1. Concentrations of genomic DNA extracted from Venere MV mud breccia push cores collected near the summit, ordered by increasing distance downslope from top to bottom.** Points shown in red were below the detection limit of the fluorometer and are plotted as values of zero, though concentrations below detection could be as high as 1.7 ng/g.

**Figure S2. Percent abundances of OTUs in Venere MV summit samples that differed across (A) older or younger summit mud flow deposits, (B) methane concentrations, and (C) chloride concentrations.** Only OTUs that were >1% in abundance among groups being compared were considered. Significance was determined at the α=0.05 confidence level with a Bonferroni correction applied for the number of pairwise comparisons (23-26, depending on treatment). Error bars represent standard errors.

**Figure S3. Heatmap of log-transformed p-values from COG category abundance comparisons between mud volcano metagenomes and those from other marine subsurface ecosystems.** Values shaded in blue indicate higher abundances in mud volcanoes, while red values are higher in other ecosystems. Statistically significant comparisons are indicated by values over or under 2.6 (α=0.05 with Bonferroni correction for multiple pairwise comparisons).

| taxon | avg % among samples | avg % among blanks | % higher in blanks | p-value of difference | max % in any non-blank | | max % in any blank |
| --- | --- | --- | --- | --- | --- | --- | --- |
| Comamonadaceae (uncl). | 0.621 | 15.566 | 14.945 | 1.019E-09 | | 16.575 | 34.304 |
| Neisseria (uncultured) | 0.014 | 11.208 | 11.194 | 2.317E-07 | | 0.275 | 33.623 |
| Corynebacteriaceae (uncl). | 0.177 | 9.709 | 9.532 | 4.695E-12 | | 4.706 | 21.807 |
| Enhydrobacter | 0.136 | 8.499 | 8.363 | 5.396E-07 | | 5.917 | 25.184 |
| Truepera | 0.228 | 5.169 | 4.941 | 5.140E-04 | | 14.365 | 15.508 |
| Halomonadaceae (uncl). | 0.202 | 4.880 | 4.677 | 1.872E-05 | | 6.071 | 14.639 |
| Fibrobacterales FD035 (gen). | 0.021 | 4.598 | 4.577 | 2.620E-07 | | 0.621 | 13.793 |
| Sulfurovum | 0.410 | 3.762 | 3.352 | 4.717E-03 | | 12.581 | 11.285 |
| Phycisphaerae MSBL9 (gen). | 0.185 | 3.553 | 3.368 | 5.471E-04 | | 9.823 | 10.658 |
| Pelagibius | 0.003 | 1.672 | 1.669 | 2.379E-07 | | 0.140 | 5.016 |
| Dehalococcoidia MSBL5 (gen). | 0.065 | 1.567 | 1.502 | 2.760E-06 | | 1.059 | 4.702 |
| Escherichia-Shigella | 0.071 | 1.555 | 1.484 | 5.032E-06 | | 1.429 | 4.665 |
| Micrococcus | 0.315 | 1.508 | 1.193 | 0.023 | | 3.677 | 4.525 |
| Paracoccus | 0.066 | 1.438 | 1.372 | 1.870E-05 | | 1.697 | 4.314 |
| Sphingomonadales (uncl). | 0.552 | 1.254 | 0.702 | 0.600 | | 14.894 | 3.762 |
| Alkanindiges | 0.000 | 1.149 | 1.149 | 2.245E-07 | | 0.000 | 3.448 |
| Phreatobacter | 0.028 | 0.927 | 0.899 | 6.331E-06 | | 1.146 | 2.780 |
| Sphingopyxis | 0.000 | 0.836 | 0.836 | 2.245E-07 | | 0.000 | 2.508 |
| Bradyrhizobium | 0.004 | 0.731 | 0.728 | 3.053E-07 | | 0.239 | 2.194 |
| Pseudomonas | 0.435 | 0.627 | 0.192 | 0.775 | | 6.378 | 1.881 |
| Corynebacterium | 0.279 | 0.418 | 0.139 | 0.801 | | 6.591 | 1.254 |
| Brevibacterium | 0.110 | 0.348 | 0.238 | 0.584 | | 5.882 | 1.043 |
| Methyloversatilis | 0.000 | 0.105 | 0.105 | 2.245E-07 | | 0.000 | 0.314 |

Table S1. Genera identified as contaminants and removed from downstream community analysis. Highlighted genus names identify reagent-associated contaminants reported in Salter et al (2014). Highlighted p-values correspond to genera that are higher in percent abundance among the three blanks (two from DNA extraction, and one PCR blank) as compared to 68 samples.

| Groups | R statistic | Significance level % | Possible permutations | Actual permutations | Number ≥ observed | | |
| --- | --- | --- | --- | --- | --- | --- | --- |
| All five groups | 0.171 | 0.5 | — | 999 | | 4 |  |
|  |  |  |  |  | |  |  |
| Pairwise tests |  |  |  |  | |  |  |
| site 1 seep GC, older summit flow | 0.345 | 0.1 | 736281 | 999 | | 0 |  |
| site 1 seep GC, summit center GC | 0.737 | 0.2 | 462 | 462 | | 1 |  |
| older summit flow, reference | 0.499 | 0.3 | 351 | 351 | | 1 |  |
| site 1 seep GC, younger summit flow | 0.416 | 0.8 | 18564 | 999 | | 7 |  |
| younger summit flow, reference | 0.568 | 1.1 | 91 | 91 | | 1 |  |
| summit center GC, older summit flow | 0.178 | 3.5 | 736281 | 999 | | 34 |  |
| site 1 seep GC, reference | 1 | 3.6 | 28 | 28 | | 1 |  |
| summit center GC, reference | 1 | 3.6 | 28 | 28 | | 1 |  |
| younger summit flow, summit center GC | 0.148 | 7.2 | 18564 | 999 | | 71 |  |
| younger summit flow, older summit flow | -0.011 | 51.2 | Very large | 999 | | 511 |  |
| younger flow (including summit center GC) and older summit flow | 0.08 | 3.4 | Very large | 999 | | 33 |  |

Table S2. ANOSIM test statistics comparing Venere MV microbial communities separated by groups depicted in Figure 6. Note that the Venere MV summit center is located on the younger mud breccia deposit. When summit center GC samples are grouped with other communities from the younger deposit, they are distinct from older summit communities, but when they are removed, no such difference is seen.

| Groups | R statistic | Significance level % | Possible permutations | Actual permutations | Number ≥ observed |
| --- | --- | --- | --- | --- | --- |
| All four environments | 0.559 | 0.1 | — | 999 | 0 |
|  |  |  |  |  |  |
| Pairwise tests |  |  |  |  |  |
| mud volcano, hydrothermal | 0.841 | 0.1 | 8008 | 999 | 0 |
| mud volcano, basalt | 1 | 0.1 | 1001 | 999 | 0 |
| mud volcano, sediment | 0.611 | 0.1 | 92378 | 999 | 0 |
| hydrothermal, basalt | 0.397 | 1.4 | 210 | 210 | 3 |
| hydrothermal, sediment | 0.266 | 2.1 | 5005 | 999 | 20 |
| basalt, sediment | 0.168 | 9.2 | 715 | 715 | 66 |

Table S3. ANOSIM test statistics comparing COG category abundances (normalized by percent) across several subseafloor environments, using metagenomes depicted in Figure 7.

| Name | Sample | Environment | Reference |
| --- | --- | --- | --- |
| JdFglass | Juan de Fuca glass minerals | Basalt | Smith et al, in review |
| JdFolivine | Juan de Fuca olivine minerals | Basalt | Smith et al, in review |
| JdFplagio | Juan de Fuca plagioclase minerals | Basalt | Smith et al, in review |
| JdFcrust | Juan de Fuca basaltic crust | Basalt | Stepanauskas et al 2012 |
| TMsmoker | Tahi Moana black smoker plume | Hydrothermal | Anantharaman et al 2016 |
| LCbiofilm | Lost City biofilm | Hydrothermal | Brazelton and Baross 2009 |
| GBplume | Guaymas Basin vent plume | Hydrothermal | Dick and Tebo 2010 |
| HAkolombo | Hellenic Arc Kolombo caldera mats | Hydrothermal | Oulas et al 2016 |
| HAsantorini | Hellenic Arc Santorini caldera mats | Hydrothermal | Oulas et al 2017 |
| JdFchimney | Juan de Fuca black smoker chimney | Hydrothermal | Xie et al 2011 |
| HMref | Håkon Mosby MV background reference | Mud Volcano | Ruff et al 2018 |
| HMz1_3.8 | Håkon Mosby MV flow, zone 1 | Mud Volcano | Ruff et al 2018 |
| HMz2 | Håkon Mosby MV flow, zone 2 | Mud Volcano | Ruff et al 2018 |
| HMz3_2.8 | Håkon Mosby MV flow, zone 3 | Mud Volcano | Ruff et al 2018 |
| HMz3 | Håkon Mosby MV flow, zone 3 | Mud Volcano | Ruff et al 2018 |
| HMz4_3.8 | Håkon Mosby MV flow, zone 4 | Mud Volcano | Ruff et al 2018 |
| HMz4 | Håkon Mosby MV flow, zone 4 | Mud Volcano | Ruff et al 2018 |
| Vflow2.7 | Venere MV flow, GC 19263-1 | Mud Volcano | This study |
| Vflow0.5 | Venere MV flow, GC 19263-1 | Mud Volcano | This study |
| Vsummit | Venere MV flow, PC 19242-15 | Mud Volcano | This study |
| PM1 | Peru Margin | Sediment | Biddle et al 2008 |
| PM16 | Peru Margin | Sediment | Biddle et al 2008 |
| PM32 | Peru Margin | Sediment | Biddle et al 2008 |
| PM50 | Peru Margin | Sediment | Biddle et al 2008 |
| BTB8 | Brazos Trinity Basin | Sediment | Biddle et al 2011 |
| ERB | Eel River Basin | Sediment | Hallam et al 2004 |
| S107 | Shimokita | Sediment | Kawai et al 2014 |
| S48.5 | Shimokita | Sediment | Kawai et al 2014 |
| S5 | Shimokita | Sediment | Kawai et al 2014 |

Table S4. Metagenome samples shown in Figure 7 with references.
